# Paediatric dentistry undergraduate education across dental schools in the Arabian region: a cross-sectional study

**DOI:** 10.1007/s40368-021-00656-9

**Published:** 2021-08-05

**Authors:** S. H. Al-Jundi, O. I. EI Shahawy, H. Nazzal

**Affiliations:** 1 Faculty of Dentistry, Jordan University of Science and Technology, Irbid, Jordan; 2 Department of Preventive Dentistry, Faculty of Dentistry, Future University, Cairo, Egypt; 3 Pediatric Dentistry, Hamad Dental Center, Hamad Medical Corporation, Doha, Qatar

**Keywords:** Undergraduate, Dental, Paediatric dentistry, Teaching methods

## Abstract

**Purpose:**

To assess and compare teaching of paediatric dentistry in the undergraduate curriculum among dental schools in the Arabian region.

**Methods:**

A 28-item online cross-sectional questionnaire survey was conducted of undergraduate dental programme directors in the Arab region. The survey included questions related to the programme’s content, method of instruction on specific paediatric dentistry topics as well as the director’s opinion on the level of training obtained by the undergraduate students at the end of the programme.

**Results:**

The final sample included 31 dental schools representing undergraduate programmes in ten Arabian countries (60.8% response rate). All programmes provided theoretical and practical education on communicative behaviour management techniques and caries prevention. Pulpectomy and formocresol pulpotomy were taught in the form of theoretical and practical education in 87.1% and 80.6% of the programmes, respectively. The method of education on common orthodontic topics was mainly theoretical with the exception of space maintainers. Instructions on managing trauma to permanent dentition was theoretical and practical in most programmes (61.3%).

Most respondents rated the level of training of students in behaviour management and caries prevention as good to reasonable, while only 22.6% thought that the level of training was excellent in pulp therapy.

**Conclusion:**

Variations were observed in paediatric dentistry education among undergraduate dental programmes in the Arabian region in terms of topics, instruction methods, year of introduction of paediatric dentistry education, and number of clinical sessions offered. This study establishes a framework for future paediatric dentistry curriculum development and/or improvement in the Arabian region.

## Introduction

Globally, more than 530 million children suffer from dental caries of primary teeth (World Health Organization 2020, https://www.who.int/news-room/fact-sheets/detail/oral-health. Accessed July 20, 2020), with a high prevalence of untreated dental caries in deciduous teeth especially in low- and middle-income countries (Peres et al. 2019). Dental needs among children in the Middle East remains high (Chen et al. 2019; Al Salami et al. 2018; Alhabdan et al. 2018), placing more emphasis on the need to provide undergraduate (UG) students with sufficient training in paediatric dentistry to a level that allows them to effectively treat children and to identify those needing specialist care, since undergraduate dental education is the starting point for future graduate provision of dental care for children (Rich et al. 2006; Stewart et al. 2010).

Most UG dental education in the Arabian countries follow a 5-year study plan with the first 3 years mainly dedicated to biological sciences, basic medical and dental sciences and pre-clinical dental training, while clinical training is carried out within dedicated facilities where clinical teaching is carried out through provision of dental care of patients in the community (BaqainZaid et al. 2016).

While some Arabian countries have national accreditation organisations that emphasise general guidelines of dental curricula, no guidelines exist for specific disciplines, such as paediatric dentistry, within the dental curriculum (Al-Amad et al. 2016). The lack of such guidelines is likely to lead to a vast variation in undergraduate paediatric dental training, therefore, resulting in a dental workforce of variable knowledge and experience.

Therefore, the aim of this study was to assess and compare teaching of paediatric dental subjects among undergraduate dental schools in the Arabian region. To the authors’ knowledge, this is the first study assessing the content, structure and methods used in undergraduate paediatric dentistry education in the Arabian region. This study would help establish baseline data as well as highlight possible differences in undergraduate education across the region allowing programme directors the necessary information for updating content of the paediatric dentistry curriculum, as well as providing the framework to establish general guidelines for paediatric dentistry curriculum in the region in view of the similarities in cultural practices, dental needs and frequency of professional exchange in the region.

## Materials and methods

This was a cross-sectional questionnaire survey which evaluated content, structure and methods used in undergraduate paediatric dentistry education in the Arabian region. Institutional ethical approval was obtained from Future University, Cairo, Egypt (ref number FUE.REC (10)/7-2019). A 28-item electronic questionnaire survey was developed and distributed electronically using the Bristol Online Survey tool (now known as Online Survey) to programme directors/academic members of staff of undergraduate dental schools in the Arabian region. Due to the lack of published data on the number of dental schools in the Arabian region, paediatric dental colleagues working in 13 Arabian countries were contacted to identify dental schools/programmes in their countries, in addition to the contact details of the paediatric dental programme directors/academic members of staff at these schools. Although 100 programmes were identified, the contact details of 51 programme directors/academic staff were identified through this method. Identified programme directors/academic members of staff were invited via email correspondence to complete the online questionnaire.

The questionnaire was distributed between April and September 2018 with two reminder emails sent in June 2018 and February 2019 for non-responders. A modified version of the questionnaire, used in assessing paediatric dental education of the UK’s UG students, was utilised (Grindrod et al. 2020). To ensure ease of understanding and reduction of ambiguity of questions, the questionnaire was piloted by two very experienced undergraduate dental education programme directors working in the Arabian region. The email circulation lists included 51 programme director/academic member of staff in the Arabian region. Individual follow-up with non-respondents was not possible due to the anonymity of the survey.

Information collected in the questionnaire included the following:

1. Demographics: data related to the country where the programme was held, type of organisation, whether an undergraduate dental programme was held and type of degree offered.
2. Questions related to the programme’s characteristics; such as number of students accepted per year, and number of batches graduated to date.
3. Questions directed to teaching methods of paediatric dentistry, which included laboratory and live patient-based clinical training in paediatric dentistry including year introduced and patient’s age range.
4. Education provided in core clinical paediatric dentistry topics: respondents selected either no instructions, theoretical only instructions, theoretical and practical or not sure to indicate training in the following clinical topics:
  - Behaviour management
  - Caries prevention
  - Pulp therapy for primary teeth
  - Pulp therapy for immature permanent teeth
  - Interceptive orthodontics
  - Management of dental trauma
  - Restorative techniques for primary teeth
  - Management of medically compromised children and special needs population.
5. Opinion of the programme director/academic member of staff: on adequacy of training of undergraduate students in the above-mentioned topics.

Data were coded and analysed using Microsoft Excel. Descriptive statistics (frequency and percentage for categorical data) were used to summarise the responses of survey participants.

## Results

Of the 51 paediatric dentistry programme directors/academic member of staff contacted, 31 responded representing ten Arabian countries, giving a response rate of 60.8% (Table 1).

**Table 1:** Dental schools identified and contacted in the Arabian region, showing individual and overall response rates

Country	Universities/programmes			Response rate (%)
	Identified *n*	Contacted *n*	Responded *n*	
Algeria	NC	NA	NA	NA
Bahrain	0	0	0	0.0
Comoros	NC	NA	NA	NA
Djibouti	NC	NA	NA	NA
Egypt	28	13	11	84.6
Iraq	13	3	3	100.0
Jordan	2	2	2	100.0
Kuwait	1	1	1	100.0
Lebanon	4	2	1	50.0
Libya	3	3	2	66.7
Mauritania	NC	NA	NA	NA
Morocco	NC	NA	NA	NA
Oman	NC	NA	NA	NA
Palestine Territories	2	2	2	100.0
Qatar	0	0	0	0.0
Saudi Arabia	23	18	2	11.1
Somalia	NC	NA	NA	NA
Sudan	10	3	3	100.0
Syria	10	0	0	0.0
Tunisia	NC	NA	NA	NA
U.A.E	4	4	4	100.0
Yemen	NC	NA	NA	NA
Total (%)	100	51	31	60.8%

*NC* no contacts available to identify number of universities or programmes, *NA* not applicable

Most schools (74%) offer a 5-year Bachelor of Dental Surgery (BDS) degree, while the number of undergraduate students per batch ranged from less than 100 to more than 300 students (Table 2).

**Table 2:** Characteristics of the dental programmes offered by dental schools in the sample

Programme		*N* (%)
Type of undergraduate programme degrees offered	5-year DDS	3 (9)
	5-year BDS	23 (74)
	6-year BDS	2 (6.4)
	Other	3 (9)
	Total	31 (100)
Number of batches of graduates of undergraduate programmes	0	2 (6.4)
	Less than 5	4 (12.9)
	5–10	4 (12.9)
	More than 10	21 (67.8)
	Total	31 (100)
Number of undergraduates per batch	Less than 100	10 (32.2)
	100–200	11 (35.4)
	200–300	2 (6.4)
	More than 300	8 (25.8)
	Total	31

*BDS* Bachelor of Dental Surgery, *DDS* Doctor of Dental Surgery

The details of the programme’s laboratory- and patient-based clinical training in paediatric dentistry are represented in Table 3. Almost half of the programmes start theoretical instructions at the 4th year, preclinical (laboratory/virtual) practical instructions in paediatric dentistry were offered by most programmes in the sample, and more than half (58%) started clinical instructions in paediatric dentistry during the 5th year, (Table 3). The number of paediatric clinical (3-h-long sessions) ranged from one every other week to six per week (12.9%, 3.2%), respectively. Slightly more than half of the programmes allow students to treat patients less than 5 years of age in the undergraduate clinics, and most respondents (67.8%) indicated that the upper age limit for patients seen in the undergraduate clinics was 12–15 years (Table 3).

**Table 3:** Characteristics of the paediatric dentistry curriculum offered by dental schools in the sample

		*N* (%)
Year of start of paediatric dentistry theoretical instructions	3rd year	6 (19.3)
	4th year	15 (48.8)
	5th year	9 (29)
	6th year	1 (3.2)
	Total	31 (100)
Year of start of paediatric dentistry preclinical instructions	No preclinical instructions	3 (9.6)
	2nd year	1 (3.2)
	3rd year	10 (32.20
	4th year	10 (32.2)
	5th year	6 (19.3)
	6th year	1 (3.2)
	Total	31 (100)
Year of start of clinical instructions	4th year	12 (38.7)
	5th year	18 (58)
	6th year	1 (3.2)
	Total	31
Number of paediatric dentistry sessions (3-h session)	1every other week	4 (12.9)
	1 per week	17 (54.3)
	2 per week	3 (9.7)
	3 per week	1 (3.2)
	4 per week	3 (9.7)
	5 per week	1 (3.2)
	6 per week	1 (3.2)
	Other*	1 (3.2)
	Total	31 (100)
Patients’ age-lower limit	< 5 years	17 (54.8)
	> 5 years	14 (45.2)
Patients’ age-upper limit	Up to 12 years	10 (32.2)
	12–15 years	21 (67.8)

*One 4-h session per week

**Fig. 1:**
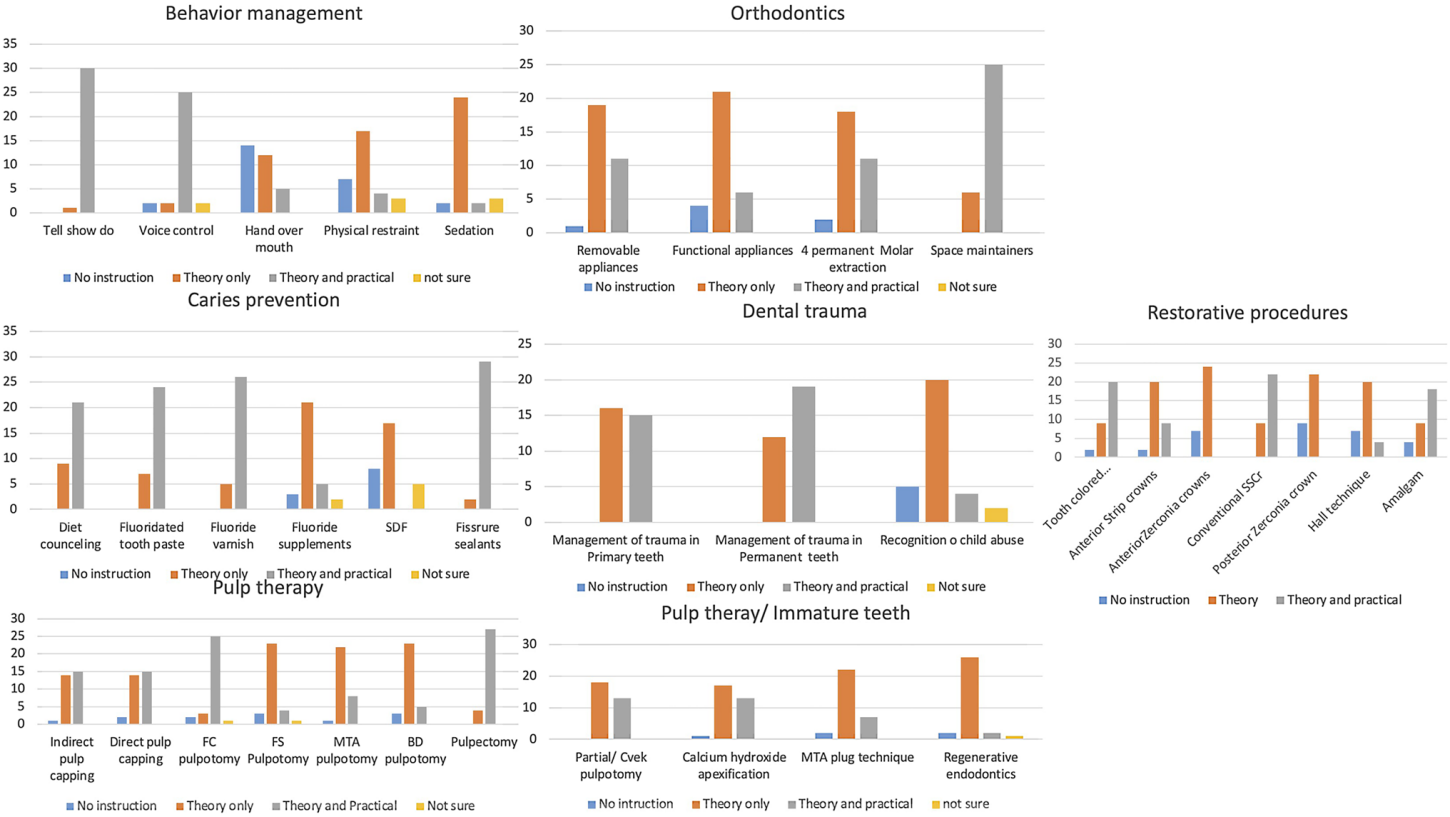
Multipanel bar chart showing education/training provided in core clinical paediatric dentistry topics delivered by dental schools in the sample

The following represent participants’ responses with regard to instruction/education in core clinical paediatric dentistry topics (Fig. 1):

a Behaviour management: All programmes provided instructions on tell-show-do (TSD) technique, mainly in the form of theoretical and practical instructions (96.7%). Voice control was the second most taught technique, with 80.6% of the programmes providing theoretical and practical instructions on this technique. On the other hand, almost half of the respondents indicated that their programmes did not provide any instructions on hand over mouth (HOM) technique. Instructions on sedation and physical restraint were mainly theoretical in 77% and 54.8% of the surveyed programmes, respectively.
b Caries prevention: Theoretical education on caries prevention was delivered by all programmes; patient-based clinical training on fissure sealant, fluoride varnish, toothpaste use and diet counselling was delivered by 93.5%. 83.8%,77.4% and 67.7%, respectively. The main method of instruction on silver diamine fluoride (SDF) and fluoride supplements was theoretical (54.8%, 67.7%, respectively), with eight respondents indicating that their programmes did not give any education on the use of SDF.
c Pulp therapy for primary teeth: Most programmes provided education on commonly practised pulp therapy techniques. While pulpectomy and formocresol pulpotomy were mostly taught in the form of theoretical and clinical instructions by 27 and 25 programmes, respectively, only theoretical education on MTA and biodentine pulpotomy techniques was delivered by most programmes (71%, 74.2%, respectively). The instructions on ferric sulphate (FS) pulpotomy were mainly theoretical (74.2%).
d Interceptive orthodontics: Most programmes used theoretical instructions only to teach common orthodontic topics with the exception of space maintainers, where theoretical and clinical instructions were used by 25 programmes (80.6%). Removable appliances and first permanent molar extraction were delivered as theoretical and practical instructions by 11 (35.5%) programmes.
e Management of dental trauma: All respondents reported that their programmes provided instructions on management of dental trauma in primary and permanent dentitions. The method of education was almost equally divided between theoretical only and theoretical and practical instructions for trauma in the primary dentition, while instructions on managing trauma in the permanent dentition was theoretical and practical in most programmes (61.3%). Non-accidental injury recognition was not taught in 16.1% of the surveyed programmes, and the main instruction method on non-accidental injury recognition was theoretical (64.5%). Three respondents were not sure if their programme provided instructions on non-accidental injury recognition.
f Pulp therapy for immature permanent teeth: Partial pulpotomy instructions were in the form of theoretical and practical education in 13 (41.9%) of the programmes, while those of MTA plug technique and regenerative endodontics were theoretical only (71%, 84%, respectively). Slightly more than half of the programmes delivered only theoretical instructions on the use of calcium hydroxide apexification.
g Restorative procedures for primary teeth: Most programmes provided theoretical and practical instructions on tooth coloured restorations, conventional stainless steel crowns (SSCrs) and amalgam restorations, (64.7%, 82.3%, 58.8%, respectively). The instruction method on more advanced restorative procedures such as zirconia anterior and posterior crowns was mainly theoretical (76.4%, 70.5%, respectively). Interestingly, most programmes (64.7%) provided theoretical instructions on Hall technique for placement of SSCrs.
h Management of medically compromised children and children with special needs: All surveyed programmes taught students basic management of medically compromised children and children with special needs. However, detailed education on the management of medically compromised children and children with special needs was given by 45.2% and 41.2% of the programmes, respectively.

**Fig. 2:**
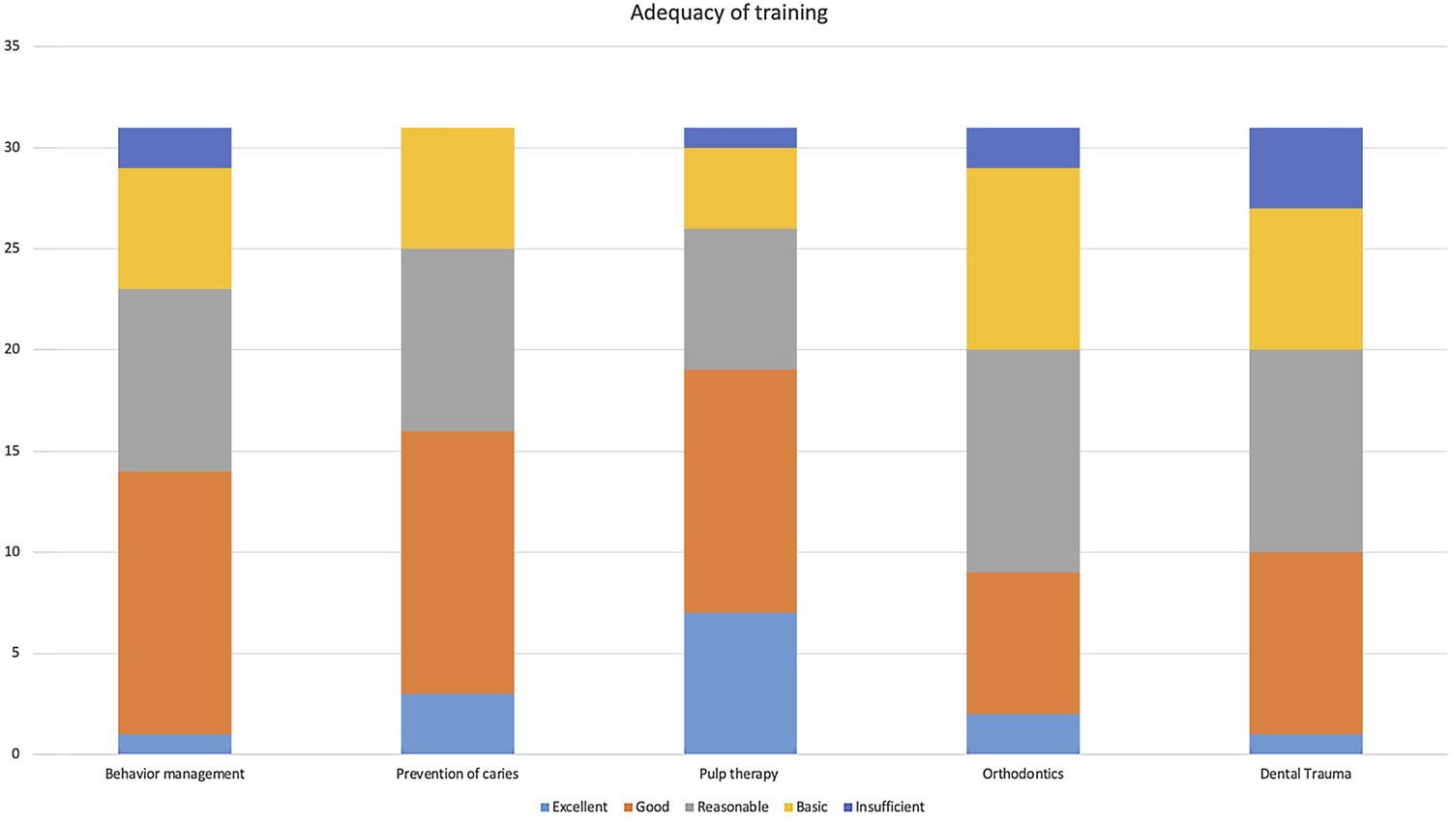
Opinion of instructors on adequacy of undergraduate training in different paediatric dentistry topics

Overall, most respondents rated the level of training of their students in behaviour management and prevention as good to reasonable. Of interest, only seven (22.6%) respondents thought that the level of training was excellent in pulp therapy, nine (29%) respondents rated their student’s training in orthodontics as basic and four (12.9%) thought that their students had insufficient experience in dental trauma management (Fig. 2).

## Discussion

This exploratory survey aimed to assess content and methods used in paediatric dentistry education of UG students in the Arabian region. The results of this survey indicated great disparities in paediatric dentistry education in terms of subjects taught, teaching methods and number of clinical sessions. Such lack of uniformity may be related to the absence of specific guidelines for paediatric dentistry undergraduate education in most of the surveyed countries (Al-Madi et al. 2018).

Despite the lack of published data on the number of dental schools in the Arabian region, the use of personal contacts allowed identification of dental schools in 11 Arabian countries and a good response rate of 60.8%.

Most surveyed programmes reported initial theoretical education, followed by preclinical (laboratory) training before commencement of patient-based clinical training. This training approach is an integral component in health professions’ education to determine students’ capabilities and implement the various domains of learning that will eventually define competent practice (Albino et al. 2008). During clinical training, students are required to develop their skills beyond the well-defined problems of the classroom to a more complex environment (Boyd 2008); therefore, preclinical laboratory training would help students bridge this gap.

There were great variations in the age of patients treated by undergraduate students across schools in the Arabian region. Interestingly, almost half of the respondents indicated that their undergraduate students treat children under the age of 5 years. This might be associated with the difficulties in providing undergraduate students with the necessary confidence in treating young preschool children. In addition, the caries experience of children at participating countries (Chen et al. 2019; Al Salami et al. 2018; Alhabdan et al. 2018) might have influenced the age range of patients at such undergraduate programmes. A relationship between the age of children treated by undergraduate students and caries level has been shown in studies in the USA and Europe (McTigue, Lee 1983; Ripa 1986; Posnick, Lanier 1989; Rodd 1994; Walker et al. 1999).

The results of this study indicated that important core topics such as communicative behaviour management (BM) techniques, prevention and pulp therapy were taught in the form of theoretical as well as clinic-based practical sessions. Dental education should take a patient-centred approach (Eriksen et al. 2008), whereby undergraduates receive competence-based training on paediatric dental patients to be able to confidently treat children in their future practices (Lekic et al. 2005). In addition, this approach is in line with published guidelines on paediatric dental undergraduate education (European Academy of Paediatric Dentistry 1997).

Most programmes provided both theoretical and practical training on communicative BM techniques with almost half not including advanced BM techniques such as hand over mouth exercise (HOME). Similar findings were reported in US dental schools where HOME is not taught in the clinic by 88% of responding programmes, while more than 75% of dental students received at least one hands-on experience with communicative BM techniques (Adair et al. 2004). This is also in agreement with international guidelines discouraging the use of HOME due to the possible psychological impact on children and medico legal implications (American Academy of Pediatric Dentistry 2019–2020.; Nunn et al. 2008). Similarly, physical restraint and pharmacologic BM techniques are considered specialised techniques; therefore, no practical training is offered by most programmes in our study. Very few programmes in the USA expected students to develop clinical proficiency with immobilisation and sedation techniques (Adair et al. 2004). In a recent survey of UK undergraduate dental programmes, BM techniques such as HOME and clinical holding were mostly delivered for knowledge rather than practice, while pharmacological techniques such as inhalation sedation and general anaesthesia involved a clinical training component (Grindrod et al. 2020). Such differences might be related to the availability of inhalation sedation and general anaesthesia for children in the community healthcare and hospital settings under the national health system in the UK.

Generally, preventive procedures were taught both theoretically and practically by most programmes in the Arabian region as similarly reported by other studies (Tikhonova et al. 2018; Walker et al. 1999). The most practised caries prevention method in our study was fissure sealant, a finding similar to that of a study in Jordan (Sonbol et al. 2012). This is likely a result of the high evidence of the success of such technique in caries prevention (Ahovuo‐Saloranta et al. 2017), its cost-effectiveness (Mitchell, Murray 1989) and its use as an introduction to dental procedures (Ahovuo-Saloranta et al. 2013).

Pulp therapy as a core topic in paediatric dentistry was included in all programmes, both theoretically and practically. Interestingly, formocresol (FC) pulpotomy was taught, both theoretically and practically, by most programmes, dissimilar to the curricula of many European schools where FC usage has declined considerably (Monteiro et al. 2017) and US dental schools are moving away from FC use in favour of ferric sulphate (Dunston, Coll 2008). Teaching of FC pulpotomy by most Arabian dental schools may be justified by its low price compared to other medicaments. Although the carcinogenic potential of FC in humans is a concern, it is not a potent human carcinogen under low exposure conditions and still used worldwide (Milnes 2008).

On the other hand, MTA and biodentine pulpotomy techniques were only theoretically taught in most of the programmes in our survey, which may be attributed to the high cost of the materials. MTA pulpotomy was the most common pulp therapy medicament taught by European dental schools (Monteiro et al. 2017). The observed variety in pulp therapy techniques in this study further emphasises the need for a consensus on the most appropriate pulp therapy techniques to be taught by universities in the Arabian region. Pulpectomy was also taught by most dental schools in our survey, similar to the findings in US and UK dental schools (Dunston, Coll 2008; Grindrod et al. 2020).

Pulp therapy for permanent teeth in the form of partial pulpotomy, pulpotomy, MTA plug and regeneration were all included in all programmes. While some programmes provided practical training on partial pulpotomy, apical plug and regeneration were mainly delivered theoretically. Calcium hydroxide apexification was still part of the programmes in spite of being recently discouraged (Duggal et al. 2017) which raises a concern about the frequent updates of the offered programmes.

With respect to orthodontic education within the paediatric dentistry curriculum, variations between programmes in terms of content and methods of instructions on various orthodontic topics have been observed. Theoretical and practical education of space maintainer use is taught by most schools surveyed, which is in line with a study in Jordan where 73% of students received hands-on experience (Sonbol et al. 2012). This is also in line with the European framework for undergraduate education in paediatric dentistry, which recommends a level of knowledge of preventive and interceptive orthodontics including the design and use of appliances for space maintenance, correction of dental crossbite and single tooth movement (European Academy of Paediatric Dentistry 1997).

With regard to dental trauma management, although most programmes gave instructions on management of dental trauma, low levels of confidence were reported by instructors regarding the adequacy of training in trauma management. This finding is echoed by many studies reporting the low levels of dental students’ confidence in managing dental trauma regionally in Jordan (Sonbol et al. 2012) and Saudi Arabia (Al-Shamiri et al. 2015), and internationally in UK dental schools (Rodd et al. 2010). The European Academy of Paediatric Dentistry suggested that dental graduates should be competent in prevention of dental trauma as well as provision of emergency treatment of acute oral and dental injuries and recognition of indications for referral (European Academy of Paediatric Dentistry 1997), which emphasises the need to establish common guidelines for trauma management education in paediatric dentistry curricula in the Arabian region. The availability of patients with dental trauma can be limited depending on the training centre and region; therefore, competencies in such subject should be carefully considered and delivered.

The lack of sufficient knowledge in dental trauma management was also documented in the literature among general dental practitioners (Hu et al. 2006) in the United Arab Emirates, where a general poor knowledge of different scenarios of TDIs management among GDPs was observed (Alyasi et al. 2018). It is worth mentioning that a high correlation was found between good knowledge of treatment and proper management of traumatic dental injuries (Alyasi et al. 2018), which again indicates the need for proper education in dental trauma management at the undergraduate level.

Most programmes provided theoretical only training on recognition of child abuse. The adequacy of this education needs requires further assessment, since some studies in the Arabian region documented major lack of knowledge of social indicators, signs of physical abuse and reporting mechanisms among undergraduate dental students as well as general dental practitioners (Al-Amad et al. 2016; Al-Dabaan et al. 2014).

The responses to the current survey indicated that teaching restorative procedures for primary teeth showed the least disparities among the surveyed topics, where most programmes provided theoretical and practical instructions on the common restorative procedures such as conventional prefabricated metal crowns (PMC), amalgam and tooth-coloured restorations. These results are similar to those reported in the UK, where most dental schools reported delivering theoretical and practical training (Grindrod et al. 2020). Hall technique is a non-invasive procedure in which the crown is cemented without local anaesthesia, caries excavation or tooth preparation. (Rosenblatt 2008). Although the use and preference of Hall technique as one of the treatment options for managing carious primary posterior teeth is increasing among general dental practitioners (Dean et al. 2011), only theoretical instructions were delivered by most programmes in the Arabian region. Despite the worldwide use of this technique, it remains controversial which might explain the lack of such clinical training (Hussein et al. 2020).

Despite the anonymous nature of this survey, the nature of this self-reported questionnaire might have resulted in a low level of unintentional bias. This could be addressed through future further assessment utilising more in-depth questionnaires and or qualitative assessment.

Further detailed assessment of paediatric dental curricula is needed with the aim of establishing structured curricular guidelines suitable for the Arabian region in view of the communities’ dental needs and access to care. Such guidelines should bring the content of the undergraduate paediatric dentistry curriculum in line with up to date evidence-based dentistry and advise on the best educational methods to deliver this content, and the most appropriate assessment tools, taking into consideration the need to adopt a competency-based approach (Donaldson et al. 2008).

## Conclusions

Variations were observed in teaching paediatric dentistry in the Arabian region in terms of subjects, instruction methods, year of introduction of paediatric dentistry education and number of clinical sessions offered. Despite the observed variations, most programmes emphasise on education in caries prevention, communicative behaviour management technique and pulp therapy for primary teeth.
